# SOD1 Deficiency Reveals Indirect Redox Stress Mechanisms Underlying Vanillin Toxicity in *Saccharomyces cerevisiae* Yeast

**DOI:** 10.3390/antiox14070842

**Published:** 2025-07-09

**Authors:** Sabina Bednarska, Magdalena Kwolek-Mirek, Roman Maslanka, Dominika Graboś, Gabriela Świniuch, Renata Zadrag-Tecza

**Affiliations:** Faculty of Biology and Nature Protection, University of Rzeszów, 35-601 Rzeszów, Poland; sbednarska@ur.edu.pl (S.B.); mkwolek@ur.edu.pl (M.K.-M.); rmaslanka@ur.edu.pl (R.M.);

**Keywords:** vanillin, oxidative stress, superoxide dismutase, ROS, redox homeostasis, *Saccharomyces cerevisiae*

## Abstract

Vanillin is a compound of great utility, and its production is, among others, based on using microorganisms such as *Saccharomyces cerevisiae* yeast. The effect of vanillin on cells is not fully understood. It has been demonstrated that vanillin induces oxidative stress; however, evidence also suggests its beneficial effects, including antioxidant and anti-inflammatory properties. For this reason, the present study was designed to elucidate the mechanism of vanillin’s action and to ascertain the extent to which its toxic effect is attributable to oxidative stress. The studies were conducted using wild-type and Δ*sod1* mutant strains. SOD1 deficiency results in cell hypersensitivity to oxidative factors, thus making the mutant strain a valuable model for investigating various aspects of oxidative stress. Based on an evaluation of cell vitality, Yap1p activation, ROS content, and glutathione and NADP(H) content, it can be concluded that oxidative stress is a secondary effect of metabolic and redox perturbations in cells rather than a direct consequence of vanillin reactivity. Furthermore, alterations observed in the redox couples GSH/GSSG and NADPH/NADP^+^ are one of the reasons for oxidative stress and suggest that vanillin may induce the utilization of NADPH for cellular needs other than antioxidant effects.

## 1. Introduction

Vanillin, an aromatic aldehyde (4-hydroxy-3-metoxybenzaldehyde), is a common flavor substance naturally occurring in *Vanilla planifolia* pods. Its usage in the food and cosmetics industries is approximately 20,000 tons per year [1]. Besides the natural origin and chemical synthesis, vanillin may be generated during lignocellulose degradation and biosynthesized using plant tissue culture, enzyme conversion, or microbial conversion [1,2,3,4]. Vanillin, as well as other by-products of lignocellulose degradation (furfural, 5-hydroxymethylfurfural, and several furanic and phenolic compounds), were found to be potent biomass fermentation inhibitors [5,6]. The need for improvement in the yield of biomass fermentation has prompted numerous efforts to enhance the tolerance of microbial strains used in biomass fermentation to inhibitory compounds, including detoxification and the development of new strains [7,8]. High demand for natural vanillin has led to a focus on genetic and metabolic engineering-assisted microbial synthesis, particularly using the yeast *Saccharomyces cerevisiae*. Engineered yeast cells that express enzymes of the shikimate pathway can produce vanillin from glucose [9]. The toxicity of the produced vanillin to host cells poses a significant challenge to achieving high yields in vanillin bioproduction. Conversely, an increasing amount of data is emerging from studies employing various models, which suggests that vanillin has antioxidant or protective effects against neurological diseases and oxidative damage [10,11].

The approaches to elucidate the mechanism of vanillin toxicity to yeast cells include vanillin uptake, vanillin biotransformation, removal of the vanillin metabolites, and the cellular response to stress generated by vanillin. Nguyen et al. have shown that vanillin is an oxidative stress-generating agent for yeast *Saccharomyces cerevisiae* cells [12], in a similar manner to another aromatic aldehyde, furfural [13]. They demonstrated that vanillin activated the Yap1 transcription factor, causing growth retardation in the Yap1-deficient strain and mitochondrial fragmentation, suggesting that vanillin induces oxidative stress in yeast cells [12]. The research presented in this work aimed to examine the role of oxidative stress in vanillin toxicity with greater breadth using the superoxide dismutase 1-deficient yeast mutant strain. Superoxide dismutases (SODs) catalyze the reaction of superoxide disproportionation to hydrogen peroxide and molecular oxygen and, thus, are crucial for oxidative stress protection. In yeast cells, two superoxide dismutase isoenzymes occur: cytosolic SOD1 (CuZnSOD) and mitochondrial SOD2 (MnSOD). A fraction of SOD1 is also distributed in the mitochondrial intermembrane space [14]. While the mitochondrial fraction of SOD1 and SOD2 is responsible for the removal of the respiratory chain-derived superoxide, cytosolic SOD1 accounts for peroxide signaling [15,16], including regulation of glycolysis and induction of NADPH production [17]. SOD1 was recently shown to prevent H_2_S cytotoxicity and the formation of other thiol-oxidizing reactive sulfur species [18]. SOD1 deficiency in yeast cells leads to hypersensitivity to several stress factors, including ROS-generating compounds such as menadione [19] and GSH-depleting compounds such as dithiopyridine [20] and aldehydes acrolein [21,22] and nonenal [23]. The lack of SOD1 protein results in the higher availability of pyridine nucleotide cofactors due to the upregulation of the pentose phosphate pathway, which implies higher activities of alcohol and aldehyde dehydrogenases [24]. Using the Δ*sod1* strain thus allows for examining the processes involved in maintaining redox balance and the impact of oxidative stress on the yeast cells after exposure to vanillin.

## 2. Materials and Methods

### 2.1. Yeast Strains and Growth Conditions

The following yeast strains were used: wild-type SP4 *MAT*α *leu1 arg4* [25] and Δ*sod1* mutant, isogenic to SP4, *MAT*α *leu1 arg4 sod1::natMX* [26]. For Yap1p activation observations, isogenic WT and ∆*sod1* strains expressing pRS cup1 cp-GFP-YAP1-kan plasmid encoding Yap1-GFP-tagged protein were used [27]. For Zwf1p location observations, the strain expressing Zwf1-GFP-tagged protein was used (*MAT*α *his3 leu2 met15 ura3 ZWF1-GFP::HIS3MX6*, Invitrogen Yeast GFP Clone Collection).

The yeast was cultivated in a standard liquid YPD medium (comprising 1% Yeast Extract, 1% Yeast Bacto-Peptone, and 2% glucose) on a rotary shaker at 150 rpm, at 28 °C.

For the vanillin treatment, cells from the exponential phase culture (~16 h; density approximately 5 × 10^7^ cells/mL) were centrifuged, washed twice, suspended to a final density of 10^8^ cells/mL in 100 mM phosphate buffer, pH 7.0, containing 1 mM EDTA and 0.1% glucose, and incubated with 6 mM vanillin for 3 h at 28 °C with shaking. Vanillin (CAS number 121-33-5, 99%, Sigma-Aldrich, Poznan, Poland) was dissolved in DMSO to obtain a 2 M stock solution. The control cells were incubated with the addition of an equal volume of DMSO. Following incubation, the cells were centrifuged, washed twice, and used for further analysis.

### 2.2. Cell Growth Assays

For spotting tests, the cells were diluted to 10^7^, 10^6^, 10^5^, or 10^4^ cells/mL. Aliquots (5 µL) of each suspension were inoculated on a solid YPD medium with 0, 2, 4, or 6 mM vanillin. The growth of the cells was inspected after 48 h.

The growth of cells in liquid YPD medium containing various concentrations of vanillin (2–9 mM), was monitored automatically at OD 600 nm using the Bioscreen C (Oy Growth Curves Ab Ltd., Helsinki, Finland) for 24 h (every 1 h) at 28 °C. The starting density of the yeast suspension was of 5 × 10^6^ cells/mL. The control cells were cultivated with an appropriate volume of DMSO (the same volume as the vanillin solution). The relative growth rate was calculated using the following equation: [(ln(OD)_t2_ − ln(OD)_t1_)/(t_2_ − t_1_)], where t_1_ is the earlier time point and t_2_ is the later time point of OD measurement during the logarithmic phase of growth.

The cell growth was also monitored by microscopic observation of the cells treated previously with 6 mM vanillin or DMSO as a control for 3 h and spread on solid YPD medium containing phloxine B (10 μg/mL; Sigma-Aldrich, Poznan, Poland). The growth of the cells was observed directly after inoculation and after 6 h using a Nikon Eclipse E200 microscope (Nikon, Tokyo, Japan) with a 20× lens and a DP10 digital camera.

### 2.3. Cell Viability and Vitality Assays

The cells after 3 h exposure to vanillin were stained with propidium iodide (PI, 5 μg/mL; Molecular Probes, Eugene, OR, USA) [28], incubated for 20 min, and observed using an Olympus BX-51 fluorescence microscope (Olympus, Hamburg, Germany). The number of dead cells, PI-positive, were expressed as a percentage of at least 200 cells in each of the three biological replicates.

The metabolic activity of the cells treated with vanillin was determined with FUN-1 stain (0.5 μM; Molecular Probes, Eugene, OR, USA) as previously described by [28]. The fluorescence of cell suspensions added with FUN-1 was quantified using a Tecan Infinite 200 microplate reader (Tecan Austria GmbH, Salzburg, Austria) with excitation and emission wavelengths of 480 and 500–650 nm, respectively. The metabolic activity was expressed as a red (λ = 575 nm) ratio to green (λ = 535 nm) fluorescence.

### 2.4. Determination of ROS Content

The generation of reactive oxygen species was assessed using dihydroethidine (DHET; Molecular Probes, Eugene, OR, USA). Following incubation with vanillin, cells were suspended to a density of 10^8^ cells/mL in a 100 mM sodium phosphate buffer, pH 7.0, containing 1 mM EDTA and 0.1% glucose. The cell suspensions were added with DHET (8 µM final concentration; stock solution in DMSO). The kinetics of fluorescence increase, resulting from the oxidation of the fluorogenic probe, was measured immediately after the addition of the probe using a Tecan Infinite 200 microplate reader at λ_ex_ = 518 nm, λ_em_ = 605 nm for DHET at a temperature of 28 °C. Additionally, cross-reactions between the probes and vanillin and DMSO in a blank (buffer without cells) were examined.

### 2.5. Determination of Glutathione Content

The total glutathione (sum of both GSH and GSSG) and separately GSSG levels were determined in the yeast cells with the GSH/GSSG-Glo Assay (Promega, Madison, WI, USA) according to the manufacturer’s protocol. Following vanillin treatment, the cells were centrifuged, washed twice with sterile water, suspended to a density of 5 × 10^5^ cells/mL in PBS buffer, and then used for determination purposes. Luminescence was recorded after 15 min using a Tecan Infinite 200 microplate reader. Total glutathione and GSSG concentrations were determined based on standard curves, whereas the level of GSH was calculated by subtracting the GSSG from the total glutathione concentration.

### 2.6. Determination of NADP(H) Content

Following exposure to vanillin, the cells were centrifuged, washed twice with sterile water, suspended to a density of 2 × 10^6^ cells/mL in a PBS buffer, and then used for the determination of cofactors with the NADP/NADPH-Glo Assay kit (Promega, Madison, WI, USA) according to the manufacturer’s protocol with own modifications [29]. The luminescence was recorded for 3 h using a Tecan Infinite 200 microplate reader. The results were presented as the content of individual pyridine cofactors in arbitrary units and the NADPH/NADP^+^ ratio.

### 2.7. Estimation of Gene Expression

Relative gene expression was estimated by real-time PCR with TaqMan chemistry. Following treatment with vanillin, the cells were centrifuged, washed twice with sterile water, and suspended to a density of 5 × 10^7^ cells/mL in the spheroplast buffer (1 M sorbitol, 0.1 M EDTA, 0.1% β-mercaptoethanol) containing lyticase (250 U per sample) for 45 min at 30 °C. The RNA samples were obtained using the GeneMATRIX Universal RNA Purification Kit (EURx, Gdansk, Poland) following the manufacturer’s instructions. The RNA samples were stored at −80 °C, and each was thawed only once. A total of 500 ng of RNA was treated with DNase I (Roche, Mannheim, Germany) for 60 min at 25 °C (10 U per 1 µg RNA) and then used for reverse transcription. To synthesize cDNA, the smART First Strand cDNA Synthesis Kit (EURx, Gdansk, Poland) was applied following the manufacturer’s instructions, and the samples were stored at −50 °C until required. The cDNA sample was diluted and mixed with FastStart Essential DNA Probes Master (Roche, Mannheim, Germany) and TaqMan Gene Expression Assays (Applied Biosystems, Life Technologies, Pleasanton, CA, USA). Real-time PCR was run using the Roche LightCycler 96 system (Roche Life Science; Mannheim, Germany). The *ACT1* gene was used as an internal control. The relative gene expression was calculated with the −ΔΔC_T_ method to compare the expression of the tested gene after treatment with vanillin with that of the untreated control in the wild-type strain.

### 2.8. Enzyme Assays

For protein extraction, the cells after incubation with vanillin were centrifuged, washed twice with sterile water, and suspended in cold homogenization buffer (20 mM phosphate buffer, pH 6.8, containing 1 mM EDTA, 0.2% DTT, and 1 mM PMSF). Subsequently, the biomass was disrupted with 0.5 mm glass beads in 6 cycles of 30 s with intervals for cooling the sample on ice. Following this, the samples were centrifuged (14,000× *g*, 15 min, 4 °C). Protein extracts were immediately frozen at −80 °C. Protein concentration was determined using the Bradford method.

The total activity of the PP pathway dehydrogenases (the sum of the activities of both glucose-6-phosphate dehydrogenase (Zwf1p) and 6-phosphogluconate dehydrogenase (Gnd1p and Gnd2p)) and separately the 6-phosphogluconate dehydrogenase activity were determined spectrophotometrically by measuring the rate of NADP^+^ reduction at 340 nm following the methodology proposed by Tian et al. [30] with our own modifications [29]. Consequently, the activity of Zwf1p was calculated by subtracting the activity of Gnd1p and Gnd2p from the total enzyme activity. To obtain the total dehydrogenase activity, the following reaction substrates were used: 0.2 mM NADP^+^, 0.4 mM D-glucose-6-phosphate, and 0.4 mM 6-phosphogluconate. The substrates were added to a 100 mM Tris-HCl buffer, pH 8.0, containing 1 mM MgCl_2_. The addition of 5 µL cell extract (comprising 2 mg of protein per mL) initiated the reaction. Consequently, to obtain Gnd1p and Gnd2p activity, only 0.2 mM NADP^+^ and 0.4 mM 6-phosphogluconate were used as reaction substrates. The kinetics of the absorbance increase was recorded using a Tecan Infinite 200 microplate reader at λ = 340 nm. The activity was expressed in arbitrary units.

The activity of glutathione reductase (GR) was determined by measuring the decrease in NADPH absorbance at 340 nm using a Varian Cary 50 spectrophotometer (Varian Ltd., Palo Alto, CA, USA). The reaction mixture included 50 mM phosphate buffer at pH 7.0, 0.5 mM DTPA, and 80 µM NADPH. To exclude nonspecific NADPH oxidation, the reaction mixture was incubated with a protein extract sample for 1 min before adding 2 mM GSSG (final concentration), after which the absorbance was recorded. GR activity was calculated with an extinction coefficient of 6.22 mM^−1^ cm^−1^ and expressed as U per mg protein.

### 2.9. Observation of Yap1-GFP Activation and Zwf1-GFP Location

For observations of Yap1p activation, cells expressing Yap1-GFP from an exponential phase culture (~16 h) were washed and suspended in 100 mM phosphate buffer, pH 7.0, containing 1 mM EDTA and 0.1% glucose, then added with 6 mM vanillin and stained with 4′,6-diamidino-2-phenylindole (DAPI, 2 μg/mL; Molecular Probes, Eugene, OR, USA) to visualize cell nuclei and observed using fluorescence microscope Olympus BX-51 equipped with the DP-72 digital camera and Cell^D and also cellSens Dimension software 4.2.1. To calculate the percentage of activated cells, at least 100 cells were observed and counted as activated when the fluorescence signal was visible from the nuclei of the cells, predominantly or partitioning between the cytosol and nuclei, but the nucleus was noticeable. The microscopic analysis was repeated 3 times (independent biological replicates).

To observe the location and content of Zwf1p, the cells carrying GFP-protein Zwf1-GFP from the exponential phase culture were washed, suspended in 100 mM phosphate buffer, pH 7.0, containing 1 mM EDTA and 0.1% glucose, and incubated with 6 mM vanillin for 3 h. Then, the cells were washed, and Zwf1-GFP fluorescence was observed using a fluorescence microscope, APEXVIEW APX100 (Olympus, Hamburg, Germany), with a BP470-490 filter. The microscopic images present representative results obtained from the duplicate experiment. Concurrently, the GFP fluorescence of the cell suspension was quantified using a Tecan Infinite 200 microplate reader, as previously described by [31].

### 2.10. Data Analysis

The data are presented as the mean ± SD from three biological replicates. The statistical analysis was performed using STATISTICA 13.3 software (StatSoft, Inc., Krakow, Poland). The statistical significance of the differences between the means of the samples treated with 6 mM vanillin and control samples added with DMSO only and between the tested strains was estimated with a *t*-test. The statistical significance of differences between means of samples treated with various concentrations of vanillin compared to a control group treated with the same volume of DMSO was estimated using one-way ANOVA and the Dunnett post hoc test. The homogeneity of variances was checked with the Brown–Forsythe test. Statistical significance was assigned to values with a *p*-value less than 0.05.

Ternary plot analysis, generated using Excel software, indicated the changes in ROS content, NADPH/NADP^+^, and GSH/GSSG ratios after vanillin treatment in triangular coordinates, which were normalized to restrict the range of values between 0 and 1.

## 3. Results

### 3.1. Vanillin Inhibits Cell Proliferation by Diminishing Cell Vitality

**Figure 1:**
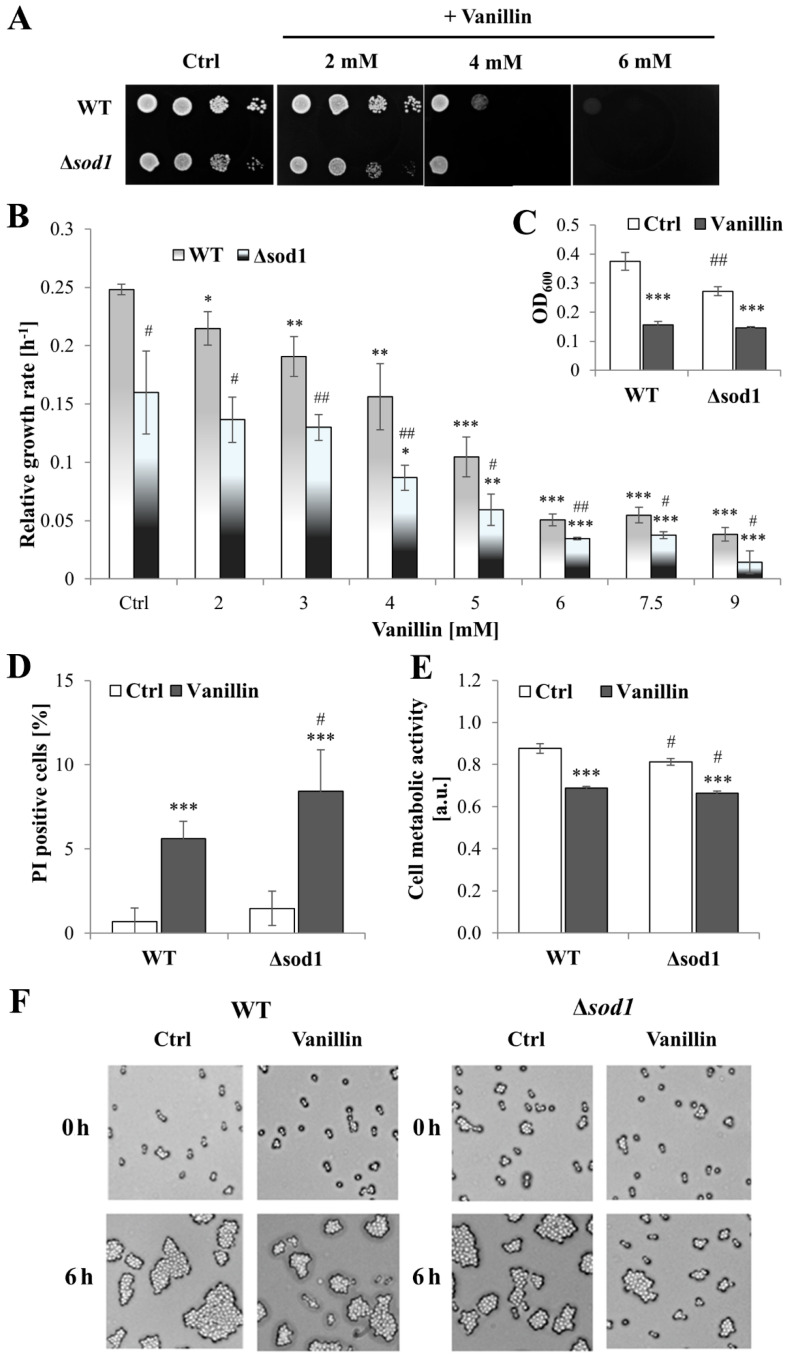
Effects of vanillin on the growth, viability, and vitality of the ∆*sod1* mutant. (**A**) The growth on solid YPD medium was examined after 48 h. The drops contain 50,000, 5000, 500, and 50 cells, respectively. (**B**) Relative growth rate in the logarithmic phase of growth. (**C**) OD_600_ measurements after 3 h of the culture with 6 mM vanillin. (**D**) The viability of the cells was estimated with propidium iodide fluorescence staining after 3 h of treatment with 6 mM vanillin. (**E**) The cell metabolic activity of yeast cells determined with FUN-1 after 3 h of treatment with 6 mM vanillin. (**F**) The viability and budding ability of yeast cells examined by microscopic analysis of cells growing on YPD agar plates with phloxine B. The pictures show representative views after the indicated time. * denotes *p* < 0.05, ** *p* < 0.01, *** *p* < 0.001 assessed *t*-test for the cells treated with vanillin vs. untreated control cells, and # denotes *p* < 0.05, ## *p* < 0.01 for the comparison between WT and ∆*sod1* strains.

To gain further insight into the physiological effects of vanillin on yeast cells and the impact of oxidative stress on toxicity, a yeast strain was used that is highly sensitive to oxidative agents due to the lack of superoxide dismutase 1 (SOD1, CuZnSOD), a primary cytosolic enzyme involved in the protection against extracellular oxidative agents [32]. The inhibitory effect of vanillin on the growth rate was confirmed to be dose-dependent (Figure 1B) and prevents growth on a solid medium (Figure 1A). In the spotting test, when vanillin was present constantly in the growth medium and the growth inspection is after 48 h, the toxicity of vanillin was visible at the concentration of 2 mM for ∆*sod1* strain and of 4 mM for both strains. The addition of vanillin at a concentration of 6 mM resulted in the complete inhibition of growth for both strains (Figure 1A). The relative growth rate reduction in the presence of vanillin (2–9 mM) in the liquid medium was not notably greater in the ∆*sod1* strain than in the wild-type strain. This was even though, in control cells without stress conditions, the growth rate of the ∆*sod1* strain was significantly reduced in comparison to the wild-type strain (Figure 1B, [26]). The observed reduction in cell growth on solid and liquid media after vanillin treatment may be attributed to either cell death and/or cell cycle arrest. Therefore, the number of dead cells was examined using propidium iodide fluorescence staining (Figure S1) after treatment of cells with 6 mM vanillin for 3 h in a buffer (3 h in the presence of vanillin in rich medium results in significant growth reduction, Figure 1C). It was found that after treatment with 6 mM vanillin, the number of dead cells increased but did not exceed 6% for the wild-type and 8% for ∆*sod1* strain (Figure 1D and Figure S1). Furthermore, observations of the cells treated with vanillin and subsequently re-inoculated into a rich solid medium revealed that, after 6 h, the cells of both strains had formed budding cell clusters, indicating that they were alive and had resumed budding. The presence of the vital dye Phloxine B in the medium confirmed that cells that did not form colonies after 6 h of re-inoculation were not necessarily dead, but their ability to reproduce was blocked. Moreover, the data for the ∆*sod1* mutant indicated slower growth of these cells but also a more extended time required to return to budding (Figure 1F). The assessment of cell vitality with fluorescent stain FUN-1 demonstrated that metabolic activity was considerably diminished in cells treated with vanillin of SOD1-deficient strain as well as of wild-type strain (Figure 1E). The impaired metabolic activity correlated with the reduced capacity of the cells to proliferate, as evidenced by the lower degree of colony development that formed after vanillin treatment compared to the control cells, which indicated that the cells undergo proliferation but with a delay (Figure 1F).

### 3.2. Vanillin Increases ROS Content and Activates Yap1p

**Figure 2:**
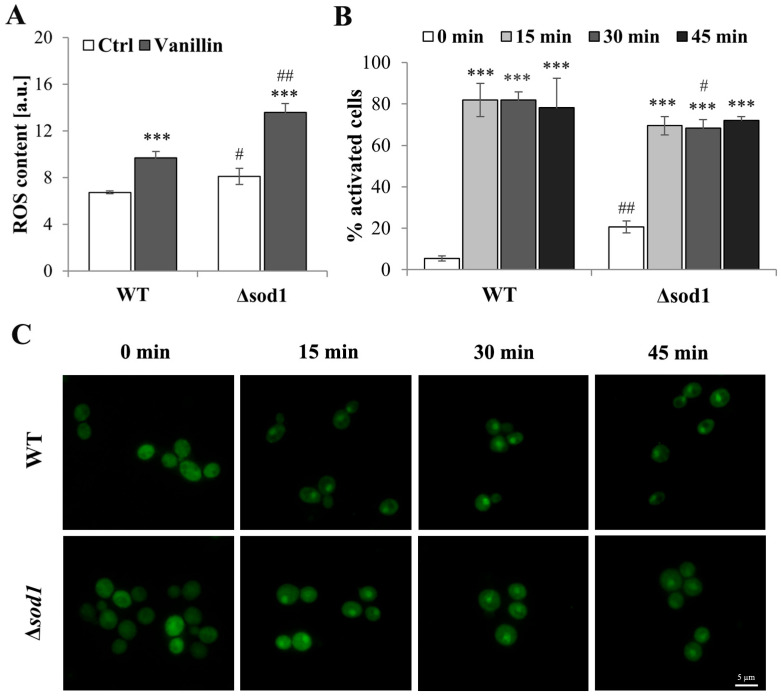
ROS content and Yap1p localization in cells treated with vanillin. (**A**) ROS content in cells treated for 3 h with 6 mM vanillin was estimated with dihydroethidine. The kinetics of fluorescence increase was measured directly after the addition of the probe. The significance of the differences between vanillin-treated samples and control cells incubated with DMSO was analyzed and *** denotes *p* < 0.001 and # denotes *p* < 0.05, ## *p* < 0.01 for the comparison between WT and ∆*sod1* strains. (**B**) The number of cells with activated Yap1p in response to 6 mM vanillin calculated at indicated time points. The cells of WT and ∆*sod1* strains expressing Yap1-GFP were observed for the indicated time and counted as activated cells when purely nuclear localization of Yap1-GFP was visible or Yap1-GFP localization was nuclear and cytosolic as well (partial activation). The significance of the differences between vanillin-treated samples and control cells incubated with DMSO was analyzed and *** denotes *p* < 0.001 and # denotes *p* < 0.05, ## *p* < 0.01 for the comparison between WT and ∆*sod1* strains. (**C**) Yap1p localization after treatment with 6 mM vanillin. The cells of WT and ∆*sod1* strains expressing Yap1-GFP were treated with vanillin and photographed for the indicated time.

To assess the occurrence of oxidative stress markers in vanillin-treated cells, the intracellular ROS content was measured with a fluorescent stain. As shown in Figure 2A, control cells from the ∆*sod1* strain showed an increased content of ROS compared to wild-type control cells. This more oxidative steady state of ∆*sod1* cells implicates greater sensitivity to oxidative stress-generating agents. Intracellular ROS levels were increased in both wild-type and mutant cells after treatment with vanillin, but to a significantly greater extent in ∆*sod1* cells (Figure 2A). These data confirmed that the disturbance of cellular homeostasis caused by vanillin had features of oxidative stress.

The state of oxidative stress is expected to induce a cellular stress response. Most oxidative stress signaling in *Saccharomyces cerevisiae* is mediated by the Yap1 transcription factor [33,34]. Induction of the Yap1-mediated oxidative stress response by vanillin was shown by Nguyen et al. [12]. For that reason, Yap1p activation after vanillin treatment in wild-type and Δ*sod1* mutant strains was analyzed (Figure 2B,C). The localization of the Yap1-GFP fusion protein was examined within 1 h of vanillin addition, and increased activation was observed with time (Figure 2C). The majority of the cells treated with vanillin revealed Yap1p activation (Figure 2B); in 70–80% of the cells, partial or complete localization of Yap1-GFP was noticed (Figure 2B; the percentage of activated cells was calculated, including all cells at least partially activated; Figure S2B). Moreover, in comparison to hydrogen peroxide, which causes complete activation (lack of cytosolic signal from Yap1-GFP, Figure S2A), 6 mM vanillin did not cause complete activation, and the response of the cells was diversified, from almost complete activation to the lack of activation (Figure 2C and Figure S2B). These results suggested that vanillin was not a strong oxidant for yeast cells. No significant differences were observed in the kinetics of Yap1p activation or duration between wild-type and ∆*sod1* strains (Figure 2C).

### 3.3. Vanillin Alters Cellular Redox State

**Figure 3:**
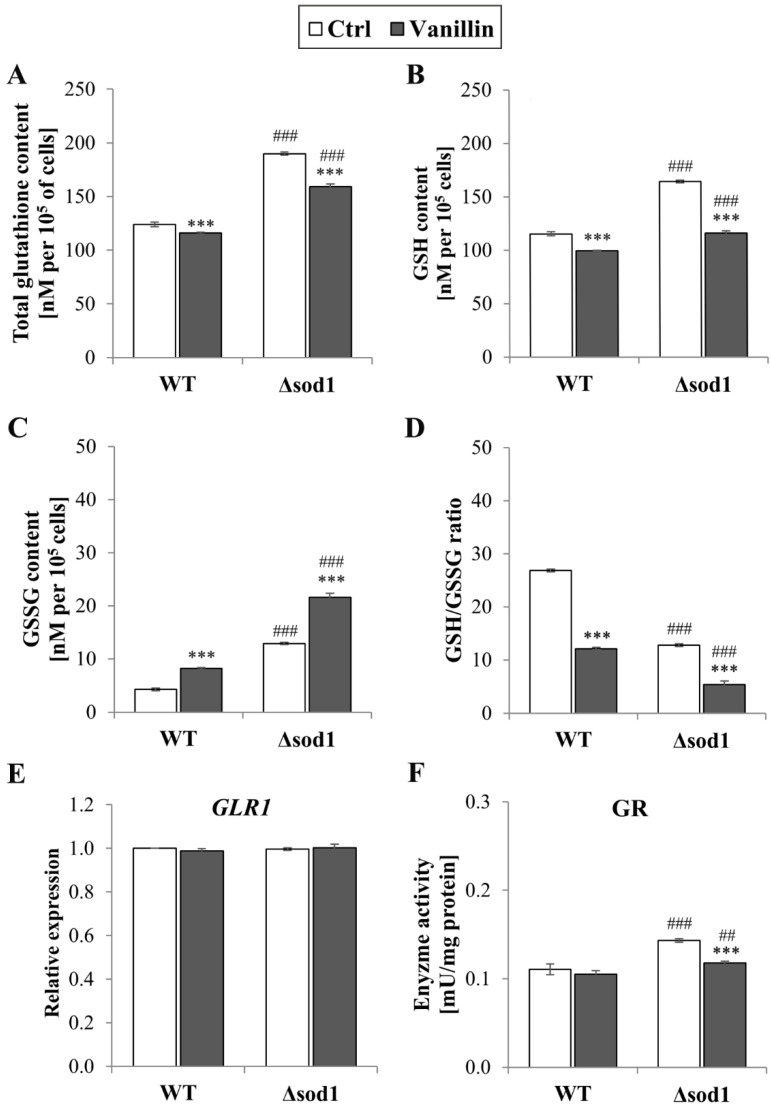
The glutathione pool in cells treated with 6 mM vanillin for 3 h: (**A**) the total glutathione content, (**B**) the reduced, (**C**) the oxidized glutathione content, and (**D**) the ratio of [GSH]/[GSSG]. (**E**) The relative expression of the *GLR1* gene was calculated using the −∆∆C_T_ method with respect to untreated wild-type cells. (**F**) The activity of glutathione reductase in whole-cell protein extracts. The significance of the differences between vanillin-treated samples and control cells incubated with DMSO was analyzed and *** denotes *p* < 0.001 and ## denotes *p* < 0.01, ### *p* < 0.001 for the comparison between WT and ∆*sod1* strains.

The maintenance of intracellular redox homeostasis depends on the glutathione system and the ability of the cells to preserve their buffering potential under stressful conditions. Therefore, the impact of vanillin treatment on the glutathione content was examined. Figure 3A–D present significant changes in total glutathione (A), reduced (B), and oxidized glutathione (C) content and the GSH/GSSG ratio (D) in both yeast strains. The depletion of reduced GSH caused by vanillin was more pronounced in ∆*sod1* than in the wild-type strain because the initial level of reduced GSH in control cells was significantly higher (about 30% compared to wild-type, Figure 3B). The decrease in reduced GSH content in vanillin-treated cells was accompanied by a significant increase in GSSG content (Figure 3C). The intracellular content of GSSG was again affected in the ∆*sod1* strain compared to the wild-type. In addition, an increase in the amount of GSSG in the ∆*sod1* strain caused by vanillin resulted in a disturbance in redox potential, expressed as the ratio of GSH/GSSG, which was significantly reduced (Figure 3D).

The imbalance in glutathione redox potential in cells exposed to vanillin was also significant in the wild-type strain. The total glutathione content, as the sum of all individual glutathione molecules, reduced and derived from glutathione disulfide, was also affected by vanillin in both strains. This suggests that glutathione oxidation to disulfide is not the only way its consumption is caused by vanillin (Figure 3A). Again, the total glutathione pool was significantly enhanced in Δ*sod1* control cells (Figure 3A). It was investigated whether the more oxidizing redox state caused by vanillin implied the induction of glutathione reductase gene expression and activity to restore the initial redox state. Glutathione reductase (GR) catalyzes the reduction of GSSG to GSH with NADPH as a cofactor. *GLR1* gene expression was unaffected after 3 h of vanillin treatment and GR activity (Figure 3E). The effect of vanillin was apparent in the case of glutathione reductase activity in the ∆*sod1* strain (Figure 3F). Again, GR activity was also affected in control ∆*sod1* cells compared to wild-type and lowered after treatment with vanillin to a level comparable with wild-type cells (Figure 3F).

**Figure 4:**
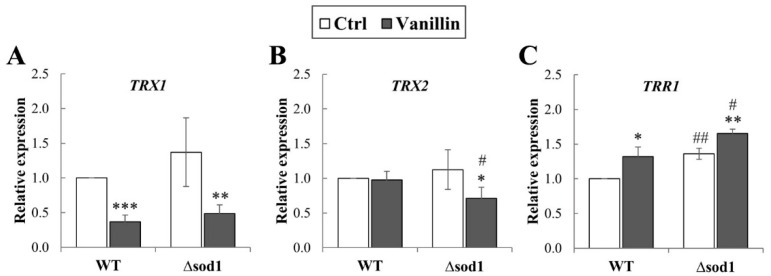
Expression of cytoplasmic thioredoxin system genes in cells treated with 6 mM vanillin for 3 h. Expression of *TRX1* (**A**), *TRX2* (**B**), and *TRR1* (**C**) genes was calculated using the −∆∆C_T_ method compared to untreated wild-type cells. The significance of the differences between vanillin-treated samples and control cells incubated with DMSO was analyzed and * denotes *p* < 0.05, ** *p* < 0.01, *** *p* < 0.001 and # denotes *p* < 0.05, ## *p* < 0.01 for the comparison between WT and ∆*sod1* strains.

Maintaining the cell redox balance requires a strict and multimodal control of the redox state of sulfhydryl groups, occurring both in glutathione (the main cellular non-enzymatic, endogenous antioxidant), but also in proteins’ Cys residues. The vital role in this thiol redox control system is played by the thioredoxin system, which consists of thioredoxins (TRXs), thioredoxin reductases (TRRs), and NADPH used as a donor. A complete cytoplasmic thioredoxin system comprises the two thioredoxins (Trx1p and Trx2p) with one thioredoxin reductase, Trr1p. Hence, it was analyzed whether the vanillin-induced alterations in redox balance are connected with changes in expression of cytoplasmic thioredoxin system genes (*TRX1*, *TRX2,* and *TRR1*). It has been found that expression of the *TRX1* gene significantly decreased in both strains after vanillin exposure (Figure 4A); the expression of *TRX2* was only reduced in the case of the Δ*sod1* mutant after vanillin exposure (Figure 4B). In contrast, the level of both genes was comparable in the control cells regardless of the strain (Figure 4A,B). The expression of the *TRR1* gene was significantly up-regulated in control ∆*sod1* cells compared to wild-type cells (Figure 4C). Furthermore, the expression of the *TRR1* gene increased similarly in both strains after exposure to vanillin (Figure 4C).

**Figure 5:**
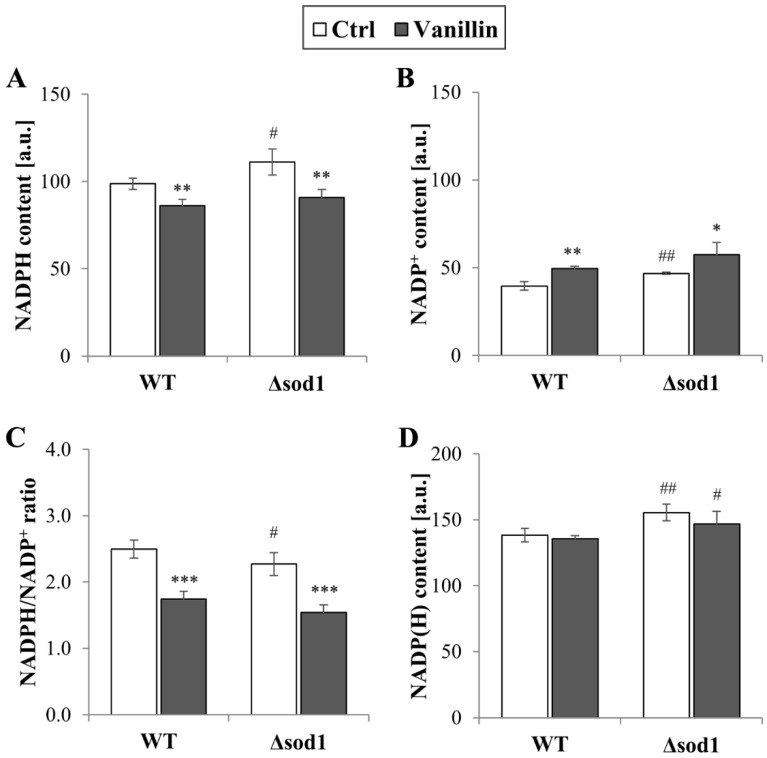
Content of NADP(H) cofactors in cells treated with 6 mM vanillin for 3 h: (**A**) NADPH, (**B**) NADP^+^, (**C**) ratio of NADPH/NADP^+^, (**D**) total pool of NADP(H) (sum of NADPH and NADP^+^). The significance of the differences between vanillin-treated samples and control cells incubated with DMSO was analyzed, and * denotes *p* < 0.05, ** *p* < 0.01, *** *p* < 0.001 and # denotes *p* < 0.05, ## *p* < 0.01 for the comparison between WT and ∆*sod1* strains.

**Figure 6:**
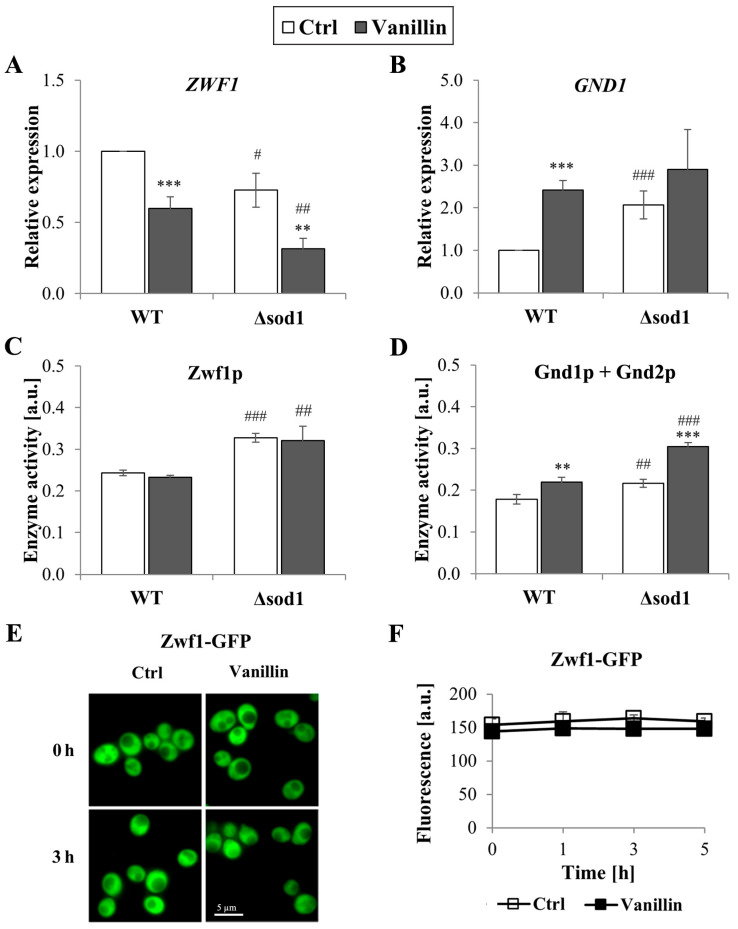
Expression and activity of pentose phosphate pathway enzymes in cells treated with 6 mM vanillin for 3 h. Expression of *ZWF1* (**A**) and *GND1* (**B**) genes was calculated using the −∆∆C_T_ method to untreated wild-type cells. Activity of PP pathway dehydrogenases (**C**) Zwf1p and (**D**) Gnd1p and Gnd2p was estimated in whole-cell extracts by measuring the increase in NADPH absorbance. Activity and location of Zwf1p in cells treated with vanillin were also assessed by observing the fluorescence of Zwf1-GFP-tagged protein (**E**) with a fluorescence microscope or by fluorescence measurements at the indicated times (**F**). The significance of the differences between vanillin-treated samples and control cells incubated with DMSO was analyzed and ** denotes *p* < 0.01, *** *p* < 0.001 and # denotes *p* < 0.05, ## *p* < 0.01, ### *p* < 0.001 for the comparison between WT and ∆*sod1* strains.

The redox homeostasis of cells is maintained by the glutathione system, supported by the thioredoxin system, both of which are dependent on the reducing power of NADPH. The levels of total and individual forms of NADP(H), as well as the ratio of reduced to oxidized forms of this cofactor, were therefore investigated. It was found that the level of NADPH decreased in cells exposed to vanillin for 3 h (a decrease of 10–20% NADPH, Figure 5A) with a concomitant increase in the level of NADP^+^ (Figure 5B). The NADPH/NADP^+^ ratio reflected these disturbances and was found appreciably diminished in vanillin-treated cells (Figure 5C). The ratio of NADPH/NADP^+^ and the content of the individual forms of the cofactor were altered again in control cells of the Δ*sod1* mutant strain (Figure 5, [24]). The total content of NADP(H) was not affected by vanillin (Figure 5D). These alterations in the redox state of the NADP(H) cofactor triggered the alterations in the expression of genes encoding dehydrogenases of the PP pathway, which is the primary source of cellular NADPH in yeast fermentative metabolism. The expression of the *ZWF1* gene encoding glucose-6-phosphate dehydrogenase was significantly reduced in both strains after exposure to vanillin (Figure 6A). In contrast, the *GND1* gene encoding 6-phosphogluconate dehydrogenase was up-regulated in both strains (Figure 6B). Determination of the activity of the respective proteins revealed that the activity of Zwf1p was not affected (Figure 6C), but the activity of Gnd1p and Gnd2p was enhanced in vanillin-treated cells (Figure 6D). The unchanged activity of Zwf1p after 3 h of vanillin treatment was confirmed by microscopic observations and quantitative measurement of Zwf1-GFP-tagged protein fluorescence (Figure 6E,F).

## 4. Discussion

The toxicity of biologically active aldehydes to yeast cells depends on their chemical structure, particularly the presence of unsaturated bonds, and can result in either growth inhibition or cell death. Molecular mechanisms of aldehyde toxicity include protein damage, formation of protein and glutathione adducts, DNA damage, depletion of the glutathione pool, secondary induction of ROS generation, and stress response [35].

Vanillin, as a phenolic aldehyde, has been shown to repress translation, form P-bodies and stress granules, and disrupt membrane integrity [36]. Many efforts have been made to find ways to alleviate the toxic effects of vanillin and other yeast fermentation inhibitors, including the overexpression of genes related to redox homeostasis [37,38] and the identification of new genes responsible for vanillin tolerance [39,40]. Our objective was to conduct a thorough investigation into the role of oxidative stress in the toxicity of vanillin in yeast cells. The involvement of oxidative stress in vanillin toxicity for yeast cells was first demonstrated by Nguyen et al. [12]. They showed that vanillin induces an oxidative stress response through activation of the Yap1 transcription factor and fragmentation of mitochondria [12]. In these studies, the oxidative stress-hypersensitive yeast strain, the Δ*sod1* mutant, was used. Lack of superoxide dismutase 1 results in increased intracellular ROS content, disturbance in glutathione homeostasis, and thus increased sensitivity to oxidative stress-generating agents. The most apparent feature of oxidative stress is the enhancement of ROS content. It was shown that treatment of cells with 6 mM vanillin considerably increases ROS content in wild-type and Δ*sod1* mutant cells (Figure 2A). This increase in ROS content, which was even more pronounced in the Δ*sod1* mutant, is an undeniable feature of oxidative stress. The increase in ROS content was observed after 3 h of incubating the cells with vanillin; in turn, shorter treatment did not lead to a significant rise in ROS levels (Figure S3). This means that the increased ROS generation is a secondary effect of cellular metabolic or redox perturbations rather than a primary effect of vanillin reactivity.

In yeast cells, the oxidative stress response is mainly mediated by transcription factor Yap1, which can be activated indirectly by modification of its reactive cysteines by Gpx3p or by direct reactive cysteines oxidation. Indirect activation is typical for peroxides, and direct activation is typical for electrophilic compounds [41], including reactive aldehydes like acrolein [21]. The activation of Yap1p in the Δ*sod1* mutant after vanillin treatment was examined (Figure 2B,C). The activation of Yap1p was observed as Yap1-GFP fluorescence accumulated in cell nuclei within 15–45 min (Figure 2C), long before the ROS increase could be detected. No difference was observed in the kinetics of Yap1p activation between the wild-type and mutant strains. The response of the cells to vanillin was very diverse, ranging from complete activation to partial to no activation (Figure 2B); even very high concentrations of vanillin did not cause Yap1p activation in each cell.

A similar effect—enhancement of ROS content after prolonged exposure (8 h) and upregulation of Yap1-dependent genes within 30–60 min—has also been shown for other aldehydes derived from lignocellulose biotransformation, furfural and 5-hydroxymethylfurfural [42]. The kinetics of Yap1p activation appear to be similar to those of menadione [41] or the thiol-reactive aldehyde, acrolein [21], in contrast to hydrogen peroxide or other peroxides, which activate Yap1p rapidly and relatively shortly [43]. This allows for the assumption that vanillin-mediated Yap1p activation is a direct reaction with Yap1p reactive cysteines rather than enzymatically mediated, as in the case of H_2_O_2_, similar to furfural and 5-hydroxymethylfurfural [42].

Cellular redox homeostasis and coping with oxidative stress require a balance between the reduced and oxidized forms of glutathione, the GSH/GSSG and NADP(H) redox couples. The connection and equilibration of the GSH/GSSG and NADPH/NADP^+^ systems have been demonstrated by several recent studies [44,45,46,47]. Glutathione is considered the main cellular source of reducing equivalents, and limiting its oxidation and GSSG formation is important. Cells possess several cooperating systems to prevent the accumulation of GSSG, but one of the most important seems to be the reduction of GSSG by glutathione reductase (GR). The GSSG reduction is accompanied by the oxidation of NADPH to NADP^+^, and glutathione reductase is an important consumer of NADPH [45]. Our results demonstrate the redox changes of the glutathione pool induced by vanillin (Figure 3). Treatment of cells for 3 h induced a significant decrease in reduced glutathione and a concomitant increase in oxidized glutathione content, affecting the glutathione redox potential expressed as the GSH/GSSG ratio (Figure 3B–D). Taking into account the total content of the two forms of glutathione, it was shown that it was significantly decreased in both strains (Figure 3A), indicating that a small amount in wild-type and about 15% of glutathione in the ∆*sod1* mutant could form adducts with vanillin, its metabolites, or with proteins modified by vanillin. This observation confirms that vanillin induces electrophilic stress like other aldehydes, but the range of this effect is rather small when compared to very reactive aldehydes, e.g., acrolein [21]. No significant alteration in the glutathione pool was observed with shorter exposure to vanillin (Figure S3). Analyzing the changes in the redox systems between the wild-type and ∆*sod1* strains under control conditions, it can be postulated that maintaining an appropriate balance between the GSH/GSSG and NADPH/NADP^+^ systems is crucial for cellular redox homeostasis. The deletion of the *SOD1* gene results in a significantly increased level of GSSG (Figure 3C). To counteract this, the following occurred: (i) glutathione reductase activity increased, even though the *GLR1* gene expression was maintained (Figure 3E,F); (ii) thioredoxin reductase (*TRR1)* gene expression is up-regulated (Figure 4C); (iii) NADPH production increased and simultaneously the level of NADP^+^ increased, indicating that the emerging NADPH pool is being consumed and can be used by GSSG-reducing systems and thioredoxin reductase (Figure 4 and Figure 5); (iv) the total glutathione and reduced glutathione levels increased (Figure 3A,B). While these activities work well enough under control conditions, the situation is entirely different when cells are exposed to vanillin. A significant increase in GSSG content was not accompanied by higher *GLR1* gene expression or higher glutathione reductase activity (Figure 3E,F). Moreover, the expression of cytoplasmic thioredoxin (*TRX1* and *TRX2*) genes decreased (Figure 4A,B), which, in light of the latest results presenting a connection between thioredoxin, Tsa1p (cytoplasmic 2-Cys peroxiredoxin), and GSSG production [48], could suggest their involvement in the development of oxidative stress. The relationship and overlap in function between the cytoplasmic thioredoxin and the glutathione/glutaredoxin system have been considered for a long time [49], although the work of Zimmmerman et al. shows directly that (i) Tsa1p is an essential source for cytosolic GSSG; (ii) Tsa1p is efficiently reduced by yeast Trx1p but not by Grx2p, and in a rate-limiting manner in a nonenzymatic way by GSH. Hence, as was suggested, the intracellular Tsa1-dependent GSSG production would require depletion or oxidation of the thioredoxin pool [48]. Related to this, a previous observation also noted that protein levels of glutathione peroxidase (Gpx2p) and cytoplasmic thioredoxin (Trx2p) did not increase in response to severe vanillin stress [12,50]. It should also be considered that the higher level of the oxidized form of glutathione in the ∆*sod1* mutant may be related not only to the presence of GSSG itself, but also to polysulfides [18,51]. This is supported by recently published data indicating that SOD1 regulates H_2_S and reactive sulfur species (RSS) such as polysulfides. Therefore, the absence of SOD1 in mutant cells may increase polysulfide levels [18]. The changes in the GSH/GSSG system are not counteracted by NADPH/NADP^+^ system changes. However, with a relatively constant pool of cofactors, the NADPH level decreases, and the NADP^+^ level increases, directly indicating its consumption (Figure 5). As the increase in *GLR1* gene expression or higher activity of glutathione reductase after vanillin exposure was not observed (Figure 3E,F), thus higher consumption of NADPH for maintaining redox balance might be connected with increased needs of thioredoxin reductase, as *TRR1* gene expression was significantly up-regulated after vanillin exposure (Figure 4C). The inadequate compensation between the GSH/GSSG and NADPH/NADP^+^ redox couples is one reason for the oxidative stress observed following vanillin treatment. Conversely, it suggests that vanillin prompts the utilization of NADPH for cellular functions beyond antioxidant activities. The decrease in NADPH levels cannot be explained by its use for anabolic needs, since the growth and proliferation of yeast cells are inhibited in the presence of vanillin (Figure 1). The observed inhibition of cell growth and proliferation is undoubtedly related to the decrease in protein synthesis caused by the repression of bulk translation activity, serving as downregulation of genes involved in ribosome biogenesis and rRNA processing [52,53]. However, inhibiting cell proliferation is not entirely a negative effect. The oxidative stress response mechanism comprises several mutually complementary pathways, whose activation incurs a high cost and consequently affects the cell’s proliferative activity. Moreover, the delay in cell cycle progression allows cells to activate an adequate repair system or break down/metabolize the xenobiotic [34]. Therefore, the use of NADPH in the cells treated with vanillin appears to be also related to the participation of NADPH in the metabolism/detoxification of this aldehyde. The main pathway of vanillin bioconversion is its reduction to the less toxic form, vanillyl alcohol, and the ratio of this conversion observed in yeast can be almost 1:1 (tested in the presence of 1 mM vanillin) [54]. As is shown by the literature data, the reduction of vanillin in yeast cells can be catalyzed by several enzymes, but the NADPH-dependent medium-chain alcohol dehydrogenases Adh6p and Adh7p play a pivotal role. Expression of Adh6p is constitutive and gradually decreases under vanillin stress, whereas expression of Adh7p is inducible and occurs under severe vanillin stress, despite the repression of bulk translational activity [52,55]. Expression of the *ADH7* gene can be activated by several transcription factors, one of which is Yap1 [52,56]. Enhanced vanillin tolerance may also be due to the effect of Gcy1p and Ypr1p, which exhibit NADPH-dependent and NAD(P)H-dependent vanillin reductase activity, respectively [39]. Additional functions in vanillin detoxification can also serve NADH-dependent aldehyde reductases (YLL056C and YNL134C), aldo-keto reductase (YJR096W), and the putative NADH-dependent alcohol dehydrogenase Bdh2p [55,56,57]. The connection between vanillin detoxification and NADPH content is supported by data investigating the deletion of the transcription factor Yrr1 or deficiency of glucose-6-phosphate dehydrogenase (Zwf1p) [50,53]. It has been shown that cells lacking Yrr1p exhibit both increased expression of the *ADH7* gene and higher NADPH-dependent vanillin reductase activity, which is associated with increased vanillin tolerance [40,53,58]. In contrast, cells lacking Zwf1p show delayed growth and inefficient reduction of vanillin in the culture [50]. Thus, faster conversion of vanillin to vanillyl alcohol and increased tolerance to vanillin in yeast cells appear to be strictly dependent on the NADPH-based detoxification system. Similarly, the crucial role of NADPH in furfural tolerance has been shown by Liu et al. [38].

Therefore, part of the cellular response to vanillin is to alter the systems that provide the reducing power of NADPH. Although yeast cells possess several cellular strategies for producing NADPH [46], the pentose phosphate pathway is still the primary source of NADPH during fermentative metabolism. The obtained results show that the protein level and activity of Zwf1p are maintained in cells treated with vanillin, even though *ZWF1* gene expression is down-regulated (Figure 6 and Figure S4). Conversely, the *GND1* gene expression and Gnd1p, Gnd2p activity were significantly up-regulated (Figure 5 and Figure S4). The obtained results are consistent with previous reports highlighting the importance of Zwf1p and Gnd1p in vanillin or furfural tolerance [38,50,58,59]. Moreover, the results showed that modulation of the expression and activity of Gnd1p seems to be an essential cellular action triggered in response to phenolic fermentation inhibitors, including vanillin (this work, [58]) and furfural [59]. Another way to supply NADPH for vanillin reduction is through the activity of aldehyde dehydrogenase 6 (Ald6p, the product of the *ALD6* gene). Our preliminary findings showed increased expression of the *ALD6* gene in cells treated with vanillin, consistent with previous reports indicating that overexpression of the *ALD6* gene enhances tolerance to vanillin, furfural, and HMF [55,56,57].

**Figure 7:**
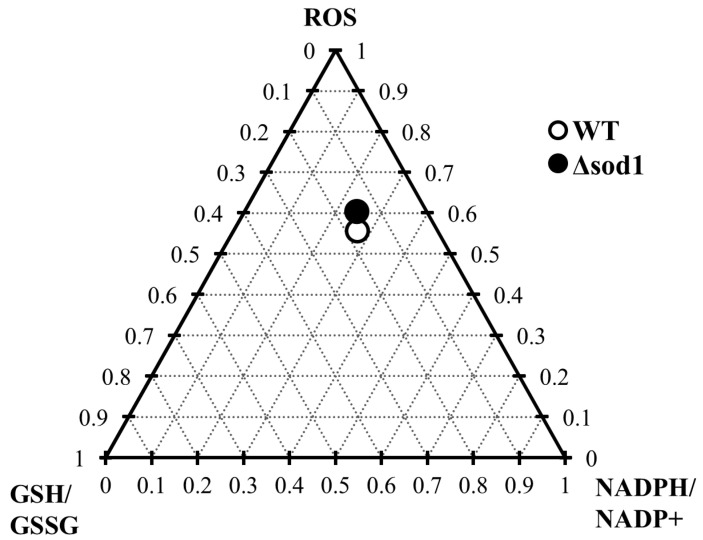
Ternary plot analysis of the influence of the ∆*sod1* mutation on the fold change in ROS content and GSH/GSSG and NADPH/NADP^+^ ratios induced by vanillin.

The depletion of the glutathione pool and the alteration in NADP(H) cofactor (Figure 3 and Figure 5) availability caused by vanillin seem to be related to diminished cellular metabolic activity (Figure 1E) and the observed inhibition of growth (Figure 1A,B), which may result from the fact that NADPH, apart from its importance for antioxidant defense systems, is also necessary for cellular biosynthetic processes. This cell vitality deterioration did not meaningfully imply cell death (Figure 1D,F), as is the case with other reactive aldehydes, e.g., acrolein [22]. This observation enables the assessment of vanillin stress as a mild electrophilic stress that leads to yeast cell cycle arrest, forcing cells to adapt to the stress conditions. To assess the impact of the SOD1 deficiency on the vanillin-induced redox perturbations, a ternary plot analysis was performed (Figure 7) with normalized ROS increase, and alterations in GSH/GSSG and NADPH/NADP^+^ ratios. The extent of vanillin-induced changes in the stress parameters is similar for wild-type and ∆*sod1* strains (Figure 7). The lack of significant differences in the fold changes of main stress markers between wild-type and stress-sensitive ∆*sod1* strains additionally supports the conclusion that oxidative stress is not the primary cause of vanillin toxicity. Our recently published data showed that deletion of the *SOD1* gene causes several alterations in the cell proteome [60], and these changes may reflect in enrichment of the redox capabilities of ∆*sod1* cells, like enhanced glutathione and pyridine cofactors content [24,61]. This redox compensation of the SOD1 deficiency may be partly responsible for the similar response of ∆*sod1* cells to factors inducing secondary electrophilic stress, like vanillin.

## 5. Conclusions

By analyzing the physiology of the cells exposed to vanillin, we can conclude that, although the markers of oxidative stress appear, their impact does not seem to be a critical factor explaining the inhibitory action of vanillin. The vanillin stress is mild and relatively a secondary electrophilic stress rather than a primary mode of vanillin’s action. We assume that alterations induced by vanillin force cells to use their available resources to detoxify this aldehyde at the expense of NADPH consumption. Although this may help reduce vanillin levels, it simultaneously reduces the pool of cellular NADPH, equivalent to the lowered abundance of overall reducing equivalents. Such alterations in the NADP(H) pool lead to a disturbance in redox homeostasis, manifested by disorders in glutathione homeostasis, lack of compensation between the GSH/GSSG and NADPH/NADP^+^ redox couples, and increased ROS generation. The results suggest that increasing the availability of NADPH cofactors appears promising in counteracting vanillin-induced inhibition of growth and fermentation in yeast cells, and it may be a direction for future research.

## Data Availability

The raw data supporting the conclusions of this article will be made available by the corresponding author upon request.

## Supplementary Materials

The following supporting information can be downloaded at: https://www.mdpi.com/article/10.3390/antiox14070842/s1, Figure S1. Effect of vanillin on the viability of the cells. The viability of the cells was estimated with propidium iodide fluorescence staining after 3 h of treatment with 6 mM vanillin. The pictures show representative views after the indicated time. Dead cells show red fluorescence (PI-positive cells). Magnification × 400. BF—brightfield; PI—propidium iodide; Figure S2: (A) Complete activation of Yap1p in the cells exposed to hydrogen peroxide (H_2_O_2_) or 9 mM vanillin. The WT cells expressing Yap1-GFP were treated with 1 mM H_2_O_2_ or 9 mM vanillin, co-stained with DAPI to visualize cell nuclei, and observed under a fluorescent microscope after 10 min incubation. (B) Diversity of Yap1p localization in WT cells in response to vanillin; a—almost complete activation; b—partial activation; c—no response, cytosolic localization of Yap1-GFP; Figure S3: ROS and glutathione content in the cells treated with vanillin. ROS content in the cells treated with 6 mM vanillin for 1 and 3 h was estimated with dihydroethidine. The kinetics of fluorescence increase was directly measured after adding the probe. Reduced (GSH) and oxidized (GSSG) glutathione content were assessed after 1 and 3 h of treatment with 6 mM vanillin with GSH/GSSG-Glo Assay. Luminescence was recorded after 15 min. The results were presented as a % of WT control. The relevance of the differences of samples treated with vanillin for control cells incubated with DMSO was analyzed and *** denotes *p* < 0.001 and # denotes *p* < 0.05, ## *p* < 0.01, ### p < 0.001 for the comparison between WT and ∆*sod1* strains; Figure S4: Expression of *ZWF1* and *GND1* genes in the cells treated with 6 mM vanillin for 1 and 3 h. The expression was calculated with the −ΔΔC_T_ method with respect to WT untreated cells. The relevance of the differences of samples treated with vanillin for control cells incubated with DMSO was analyzed and ** denotes ** *p* < 0.01, *** *p* < 0.001 and # denotes *p* < 0.05, ## *p* < 0.01, ### *p* < 0.001 for the comparison between WT and ∆*sod1* strains.
