# Supplementary material for: SOD1 Deficiency Reveals Indirect Redox Stress Mechanisms Underlying Vanillin Toxicity in Saccharomyces cerevisiae Yeast
Source: Antioxidants (Basel). 2025 Jul 9;14(7):842. doi: 10.3390/antiox14070842 (PMC12291783; doi:10.3390/antiox14070842)
Supplement: Supplementary file 1 [file antioxidants-14-00842-s001.zip › antioxidants-3654366-supplementary.pdf]

Supplementary Materials

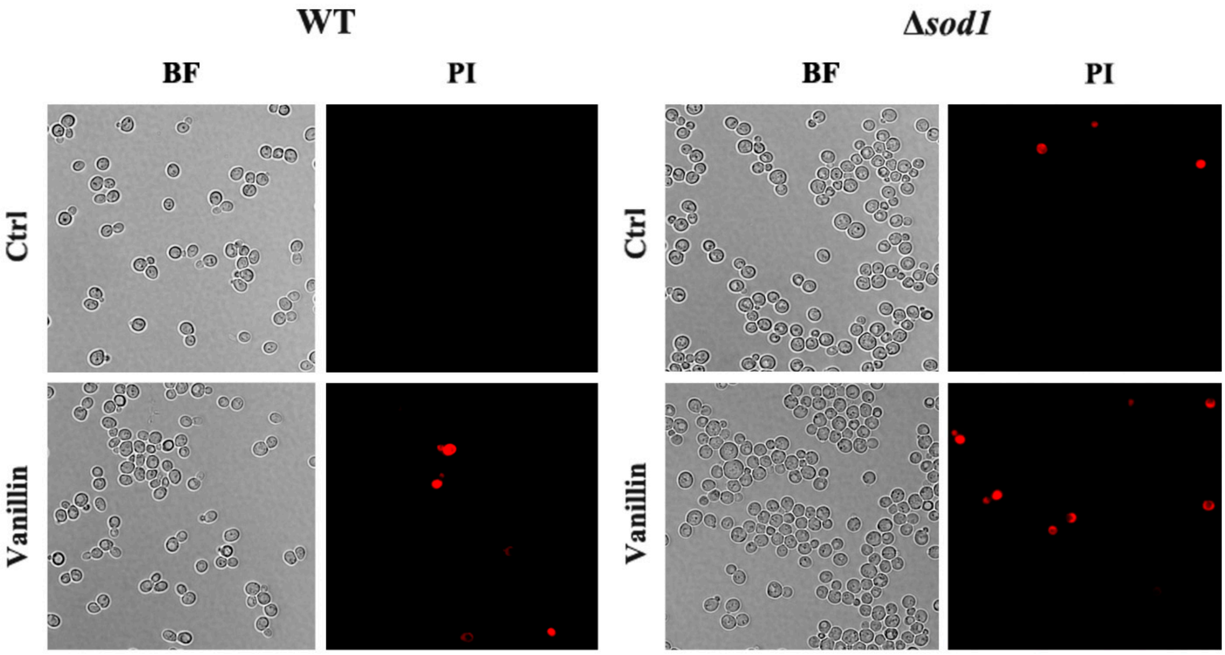

Figure S1. Effect of vanillin on the viability of the cells. The viability of the cells was estimated with propidium iodide fluorescence staining after 3 h of treatment with 6 mM vanillin. The pictures show representative views after the indicated time. Dead cells show red fluorescence (PI-positive cells). Magnification × 400. BF – brightfield; PI – propidium iodide

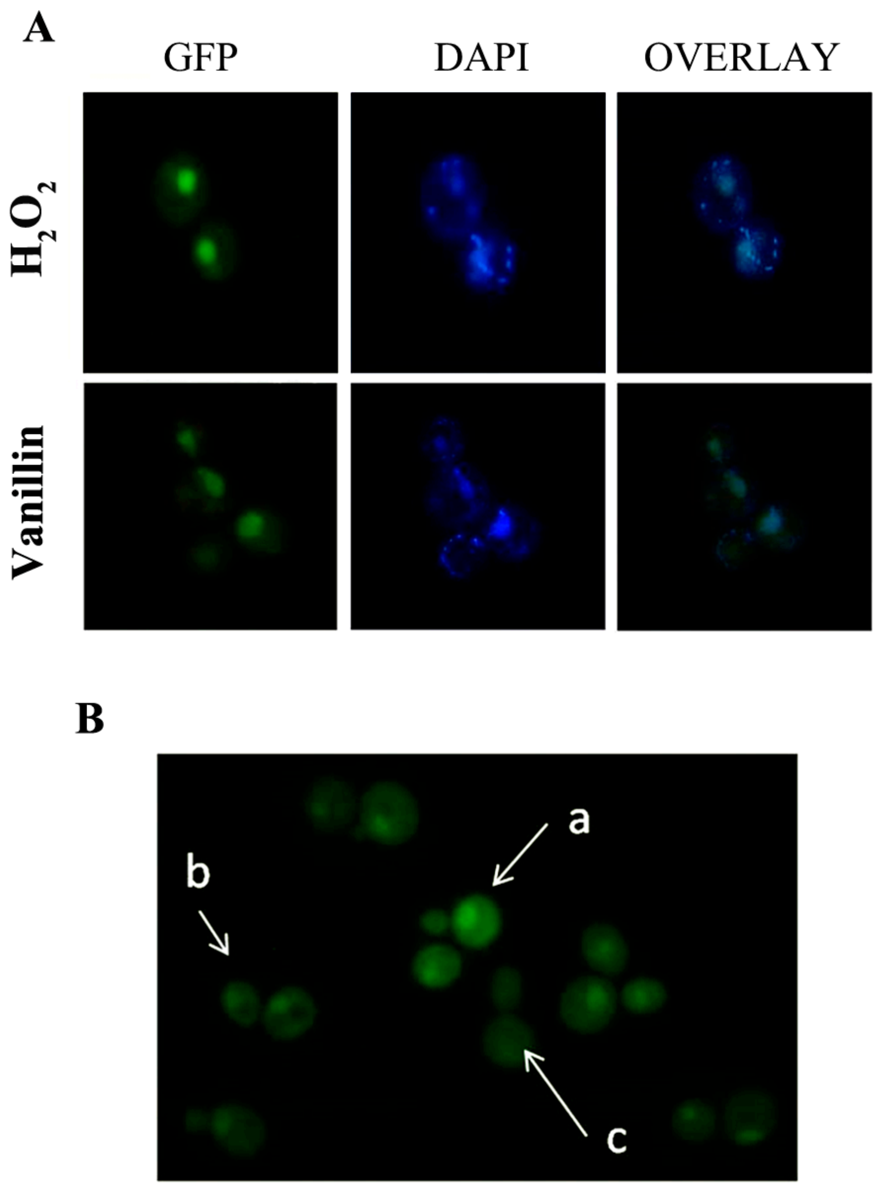

Figure S2. (A) Complete activation of Yap1p in the cells exposed to hydrogen peroxide (H_2O_2) or 9 mM vanillin. The WT cells expressing Yap1-GFP were treated with 1 mM H_2O_2 or 9 mM vanillin, co-stained with DAPI to visualize cell nuclei, and observed under a fluorescent microscope after 10 min incubation. (B) Diversity of Yap1p localization in WT cells in response to vanillin; a - almost complete activation; b - partial activation; c - no response, cytosolic localization of Yap1-GFP

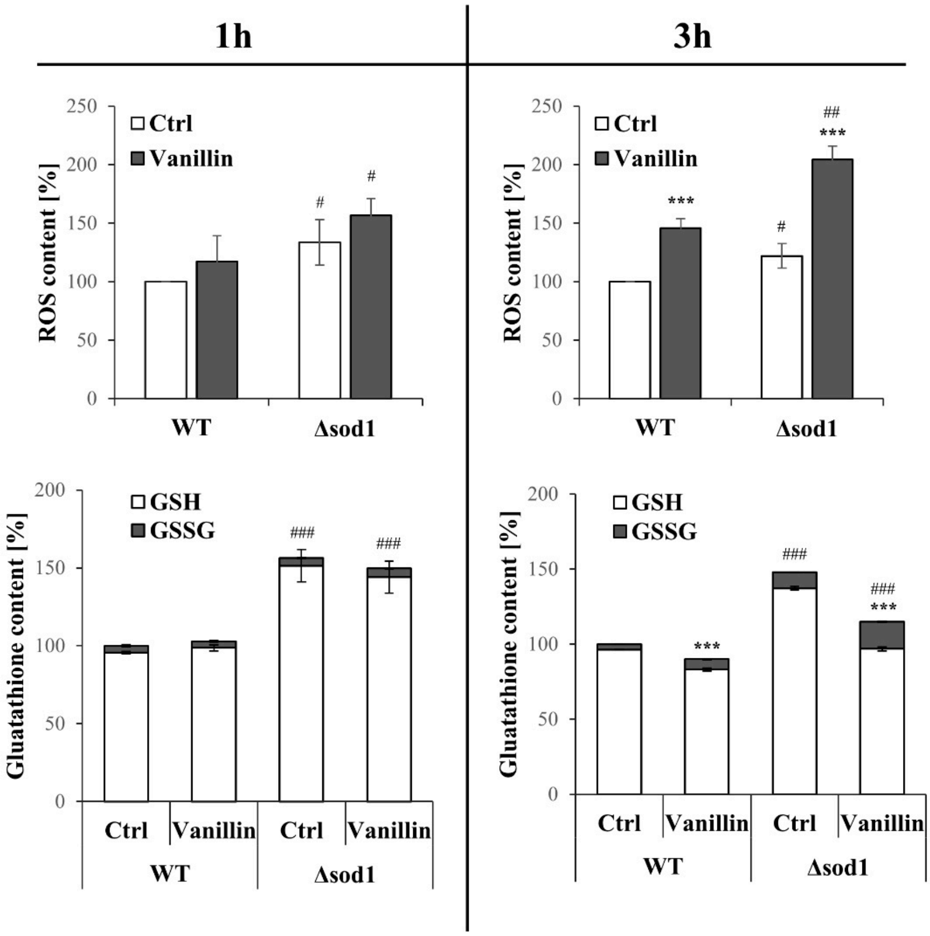

Figure S3. ROS and glutathione content in the cells treated with vanillin. ROS content in the cells treated with 6 mM vanillin for 1 and 3 h was estimated with dihydroethidine. The kinetics of fluorescence increase was directly measured after adding the probe. Reduced (GSH) and oxidized (GSSG) glutathione content were assessed after 1 and 3 h of treatment with 6 mM vanillin with GSH/GSSG-Glo Assay. Luminescence was recorded after 15 min. The results were presented as a % of WT control. The relevance of the differences of samples treated with vanillin for control cells incubated with DMSO was analyzed and *** denotes $p < 0.001$ and # denotes $p < 0.05$, ## $p < 0.01$, ### $p < 0.001$ for the comparison between WT and Δ*sod1* strains.

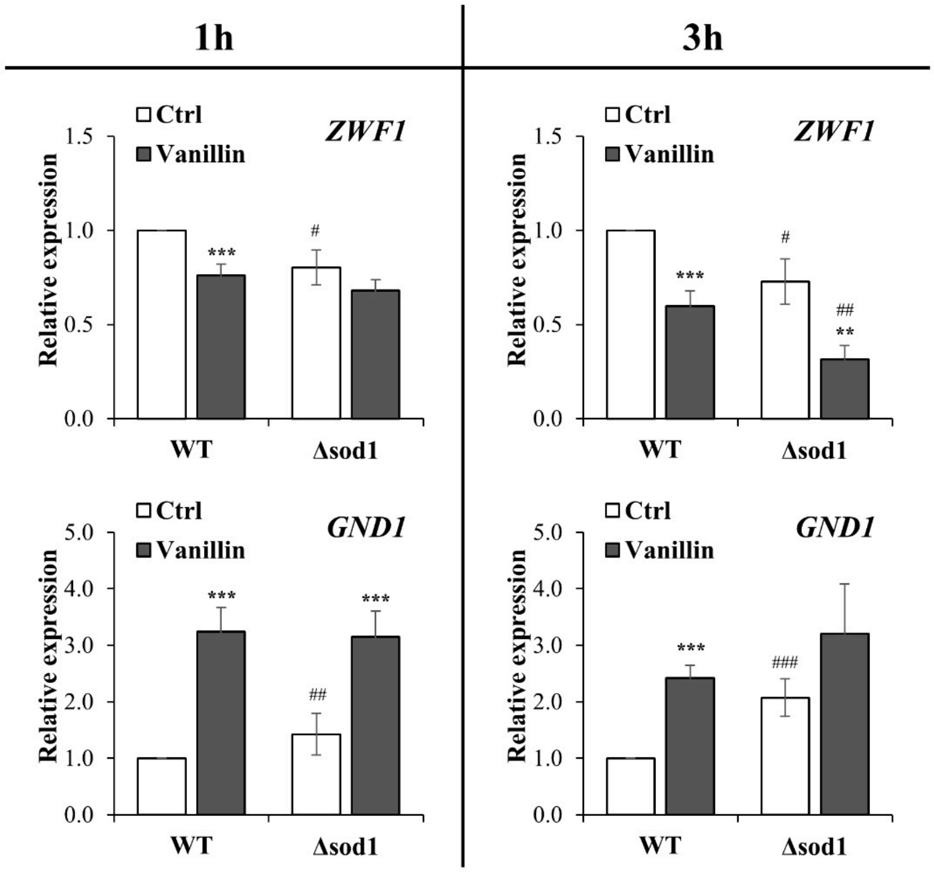

Figure S4. Expression of *ZWF1* and *GND1* genes in the cells treated with 6 mM vanillin for 1 and 3 h. The expression was calculated with the -ΔΔCT method with respect to WT untreated cells. The relevance of the differences of samples treated with vanillin for control cells incubated with DMSO was analyzed and ** denotes ** $p < 0.01$, *** $p < 0.001$ and # denotes $p < 0.05$, ## $p < 0.01$, ### $p < 0.001$ for the comparison between WT and Δ*sod1* strains.
